# In the Red: Debt, Financial Well-Being, and Retirement Preparedness Among Older Adults

**DOI:** 10.1093/geroni/igaa057.2431

**Published:** 2020-12-16

**Authors:** Julie Miller, Julie Miller

## Abstract

Over the past twenty years, total debt for Americans ages 70 and over has increased more relative to all other ages group- by 543% according to the Federal Reserve Bank of New York (2019). Higher rates of debt among older adults have been attributed to a range of factors including expanded access to consumer credit, the capability for people to borrow from 401(k) plans, increases in costs of living, and limited financial literacy, among others. Related research of adults nearing retirement age who carry debt points to delayed retirement timing, lower levels of retirement savings, and higher risk and rates of bankruptcy. This symposium introduces timely investigations of retirement planning and economic security among older adults with debt. The first presentation will provide an overview of the impact of financial hardship on health among older adults in the United States over a recent ten-year period. The second presentation will focus on over-indebtedness among pre-retirees. The third presentation will examine the role of safety net services and borrowing from retirement plans among older adults with debt, particularly among older adults of color. The fourth and final presentation will focus on student loan debt as a hurdle to near-term and long-term financial security for older women in particular. A discussant will comment on how, together, the aforementioned papers contribute to our understanding of economic wellbeing and retirement preparedness in this era of increasing longevity. The session will integrate policy and programming implications for gerontological professionals.

